# Microbial Diversity in the Phyllosphere and Rhizosphere of an Apple Orchard Managed under Prolonged “Natural Farming” Practices

**DOI:** 10.3390/microorganisms9102056

**Published:** 2021-09-29

**Authors:** Ying-Hong He, Charith Raj Adkar-Purushothama, Tsutae Ito, Asuka Shirakawa, Hideki Yamamoto, Akiko Kashiwagi, Ayumu Tatewaki, Misato Fujibayashi, Shuichi Sugiyama, Katsuhiko Yaginuma, Tomoya Akahira, Shingen Yamamoto, Seiya Tsushima, Yuko Matsushita, Teruo Sano

**Affiliations:** 1 Faculty of Agriculture and Life Science, Hirosaki University, Hirosaki 036-8561, Japan; kakuinhirosaki@yahoo.co.jp (Y.-H.H.); charith.raj.adkar.purushothama@usherbrooke.ca (C.R.A.-P.); asbkr8.516@gmail.com (A.S.); hyamhome@gmail.com (H.Y.); tatewaki.ayumu916@outlook.jp (A.T.); misato.f.9166@gmail.com (M.F.); sugi@hirosaki-u.ac.jp (S.S.); 2 RNA Group/Groupe ARN, Département de Biochimie, Université de Sherbrooke, 3201 rue Jean-Mignault, Sherbrooke, QC J1E 4K8, Canada; 3 Division of Apple Research, Institute of Fruit Tree and Tea Science (NIFTS), National Agriculture and Food Research Organization (NARO), Morioka 020-0123, Japan; denito@affrc.go.jp (T.I.); yaginuma@affrc.go.jp (K.Y.); 4 Apple Research Institute, Aomori Prefectural Industrial Technology Research Center, Kuroishi 036-0332, Japan; tomoya_akahira@aomori-itc.or.jp (T.A.); shingenome@gmail.com (S.Y.); 5 Japan Fisheries Research and Education Agency, Fisheries Technology Institute, Fisheries Engineering Division, Kamisu 314-0408, Japan; 6 National Agro-Environment Research Institute, Tsukuba 305-0856, Japan; seiya.tsushima@gmail.com (S.T.); yuh1528@gmail.com (Y.M.); 7 Faculty of Agriculture, Tokyo University of Agriculture, 1737 Funako, Atsugi 243-0034, Japan

**Keywords:** microbial diversity, phyllosphere, rhizosphere, macroarray, high-throughput sequencing, natural apple farming, suppression of disease, metagenome

## Abstract

Microbial diversity in an apple orchard cultivated with natural farming practices for over 30 years was compared with conventionally farmed orchards to analyze differences in disease suppression. In this long-term naturally farmed orchard, major apple diseases were more severe than in conventional orchards but milder than in a short-term natural farming orchard. Among major fungal species in the phyllosphere, we found that *Aureobasidium pullulans* and *Cryptococcus victoriae* were significantly less abundant in long-term natural farming, while *Cladosporium tenuissimum* predominated. However, diversity of fungal species in the phyllosphere was not necessarily the main determinant in the disease suppression observed in natural farming; instead, the maintenance of a balanced, constant selection of fungal species under a suitable predominant species such as *C. tenuissimum* seemed to be the important factors. Analysis of bacteria in the phyllosphere revealed *Pseudomonas graminis*, a potential inducer of plant defenses, predominated in long-term natural farming in August. Rhizosphere metagenome analysis showed that *Cordyceps* and *Arthrobotrys*, fungal genera are known to include insect- or nematode-infecting species, were found only in long-term natural farming. Among soil bacteria, the genus *Nitrospira* was most abundant, and its level in long-term natural farming was more than double that in the conventionally farmed orchard.

## 1. Introduction

Microbial diversity, in both the phyllosphere and the rhizosphere, is thought to influence the formation and function of microbial environments in crops [1,2,3]. Changes in the microbial community always lead to alterations of the host metagenomic environment, reflecting the ways in which pathogens attack and divert host functions and resources and the ways hosts respond to pathogen invasion [3]. Consequently, predicting how changes in microbial diversity affect the functioning of an ecosystem requires an understanding of the complex interactions among microbial species at multiple levels [4]. Thus, characterization of changes in microbial diversity under various cultural or pest-control conditions can facilitate understanding of host–parasite interactions [5,6]. Previously, the identification of pathogens that cause severe to mild disease symptoms could only be performed individually or in small groups. With the recent availability of high-throughput methods, such as DNA microarray or large-scale sequencing techniques, multiple microbial species can now be analyzed simultaneously. The diversity of microbial inhabitants, especially those living in the phyllosphere or rhizosphere of agricultural crops, can be influenced by the natural environment (temperature, rainfall, soil type, mineral content) or by human activities, including cultural practices and pest-control management (chemical fertilizers, pesticides). In turn, changes in microbial diversity are believed to play an important role in the health status and disease incidence among crops, either via competitive or antagonistic action toward pathogens or by stimulating the potential resistance and natural immunity of plants against them [7,8,9,10,11,12,13,14].

In this study, we examined an apple orchard located in Aomori, northern Japan, covering an area of around 0.4 ha and planted with around 50 apple trees, including the varieties “*Fuji*” and “*Tsugaru*”. Since the orchard was converted from conventional to natural farming more than 30 years ago, it has been managed without the use of any chemicals (insecticides, fungicides, herbicides, and fertilizers) or even any organic manure. After initial conversion to natural farming, the trees bore no apples for several years, due mainly to severe damage by pests and diseases. However, after different approaches were tried, with much effort over 10 years, the orchard began to produce a crop. An example of a natural pest-control trial involved spraying the trees with diluted edible vinegar approximately weekly, after which productivity gradually improved and stabilized [15]. At present, the orchard is not free from pests or disease; in fact, they are quite common: Monilinia blotch epidemics occur early in spring, scab fungi cause symptoms on the leaves in early to mid-summer, and Alternaria and Marssonina blotch sometimes cause premature defoliation in autumn. However, despite this, the practice of natural farming over many years has resulted in economically viable yields of apples in this commercial orchard.

Unsurprisingly, the events taking place in this orchard have not been looked upon favorably by the majority of farmers and researchers in the apple-growing industry. Even those who support the natural farming practices of the orchard and recognize its success do not accept that these methods could be broadly applied for commercial apple production. However, we were intrigued by the success of this orchard, hypothesizing that various factors such as tree vigor, the richness of microbial diversity, and soil nourishment may have converged into a stable state after so long, warranting further study. It is increasingly apparent that environmental concerns are of critical importance in the 21st century, and this is especially true in the domain of agriculture. Humanity needs to further improve productivity by taking the environment into consideration, informed by advanced farming techniques that have already been developed. Therefore, we felt it was essential to analyze and learn from what is happening in this orchard to identify positive factors worthy of future application in apple cultivation.

In previous studies, we used oligo-DNA macroarrays to monitor the major phytopathogenic and non-phytopathogenic fungi and bacteria inhabiting the phyllosphere in apple orchards (including the aforementioned prolonged “natural farming orchard” in the 2009 season [16]). Soil bacterial community diversity in naturally farmed and conventionally farmed apple orchards was also evaluated using 16S rRNA gene sequencing, and we found that many significant differences in the richness and evenness of operational taxonomic units were detectable at each taxonomic level (17). Here, integrating field investigations of major apple diseases, macroarray and metagenome analyses (next-generation sequencing) of microbial inhabitants, and traditional agar plate culture methods to monitor specific species of interest, we further analyzed differences in microbial diversity under various pest management regimes, surveying natural farming (organic) orchards in comparison with conventional orchards subject to chemical pesticide treatment. Our results reveal the microbial species characteristic of the phyllosphere and rhizosphere of an apple orchard managed for a prolonged period under natural farming conditions.

## 2. Materials and Methods

### 2.1. Apple Orchards

Natural-AK: This orchard (≈0.4 ha) is located in Hirosaki, Japan (140°25′ E 40°37′ N). The trees have been managed for over 30 years under natural farming conditions without the use of any chemical fertilizers, organic manures, chemical pesticides (fungicides and insecticides), or herbicides. The only treatment used against pests and disease was a specially formulated vinegar solution (Seisen-15, Mizkan Co., Ltd., Handa, Japan), diluted around 200–500 times, sprayed 15 times per season at weekly or biweekly intervals from early May to mid-September. During the period of our study (the 2010–2015 growing seasons), all developing apples were wrapped in paper bags from early June to the end of September to protect them from insect invasions such as peach fruit moths (*Carposina sasakii*). Weeds beneath the trees were cut twice a year, in May and September, and left onsite to serve as green manure. Three “*Fuji*” trees of about 35 years old (unknown rootstock) were selected for the analysis. Our research activities at this location were made possible through the personal kindness of the owner.

Chemical-O: This orchard (≈0.7 ha) is located in the plot neighboring Natural-AK. The trees have been managed under normal cultivation conditions whereby chemical pesticides and fungicides are sprayed 11 times in the growing season according to an intensive calendar-based pest management regime. In general, chemicals sprayed include fenbuconazole, Score MZ (difenoconazole and mancozeb), ziram, thiram, iminoctadine triacetate, Aliette-C WP (captan), Flint Flowable25 (trifloxystrobin), cyprodinil, Antracol WG (prospineb), calcium carbonate, and organic copper compounds as fungicides; and machine oil, organophosphorus compounds (e.g., chlorpyrifos), pyrethroid (e.g., cypermethrin), and neonicotinoids (e.g., clothianidin) as insecticides. Organic and inorganic fertilizers were supplied slightly differently depending on the year, which included Cosmogreen (Cosmogreen K.K., Kumamoto, Japan) that includes sewage sludge, animal/plant residue, and oyster shells, or Gold-tokugo 085 (Sunbiotic, Nagasaki, Japan) that includes rapeseed/castor oil cake, bone meal, and fish and shellfish waste. They include nitrogen (N), phosphorus (P), and potassium (K), and chemical fertilizers. The application rate (kg/ha) of N, P, and K was, for example, 45.0, 36.0, and 22.5 in 2009, and 85.0, 68.0, and 42.5 in 2010, respectively. Three “*Fuji*” trees of similar tree size (age and rootstock unknown) to Natural-AK were selected for analysis. Our research activities at this location were made possible through the personal kindness of the owner.

Natural-MAR: This orchard was established in 2009 as an experimental mimetic model of Natural-AK in a plot in the experimental fields belonging to the Apple Research Center, Morioka, Japan (E 141°09′ N 39°42′), located ca. 100 km southeast of the Natural-AK orchard. Since its inception, the trees have been managed under natural farming conditions mimicking the methods at Natural-AK. The orchard (≈0.05 ha) consists of 10 trees of two cultivars, including five “*Fuji*” and five “*Starking*”. Three “*Fuji*” trees of 35 years old on M9 rootstock were selected for analysis.

Chemical-MAR: This plot was also established in 2009 as a control for Natural-MAR in the neighboring plot. The trees have been managed under conventional cultivation conditions whereby chemical pesticides and fungicides are sprayed 11 times during the growing season according to an intensive calendar-based pest management regime, which is essentially the same as the Chemical-O orchard. The plot (≈0.05 ha) consists of nine trees of three cultivars, including three “*Fuji*”, four “*Starking*”, and two “*Shinano Gold*”. Three “*Fuji*” trees of 35 years old on M9 were selected for analysis.

Left-alone-MAR: This orchard was also established in 2009 as a control for the neighboring “Chemical-MAR” and “Natural-MAR” plots. The plot (≈0.05 ha) consists of nine trees of four cultivars, including four “*Fuji*”, three “*Starking*”, one “*Shinano Gold*”, and one “*Kiou*”. The trees in this plot were cultivated without any treatment of chemical fungicides, chemical pesticides, vinegar, chemical fertilizers, and even compost. Three “*Fuji*” trees of 35 years old on M9 were selected for analysis.

### 2.2. Disease Incidence

Regarding the major apple leaf diseases, three branches were selected from three ”*Fuji*” trees, and 100 leaves per branch (a total of 300 per tree) were inspected. The major diseases were apple scab, Alternaria blotch, Marssonina blotch, Monilinia blight, and early leaf drop, mainly caused by Marssonina blotch. The severity of the disease incident was scored by the number of spots from 1 to 5 and from 6 to 10 per leaf for apple scab, and 1 to 10, 11 to 50, and 51 to 100 for Alternaria blotch.

### 2.3. Macroarray Analysis of Microbial Diversity in the Phyllosphere

Leaf samples were collected from five orchards (Natural-AK, Chemical-O, Natural-MAR, Chemical-MAR, and Left-alone-MAR) during August and September in 2010–2015. Three trees were selected in each orchard, and from each; three leaves were collected from each of three different branches (i.e., nine leaves per tree or 27 per orchard). The nine leaves from each tree were pooled and crushed into small pieces, mixed well, put in liquid nitrogen, and an aliquot (0.5 g) was taken for DNA extraction. DNA extraction, polymerase chain reaction (PCR) amplification of fungal rDNA-ITS and bacterial 16S rDNA, DIG-labeled RNA probe preparation, and oligo-DNA macroarray hybridization were performed essentially as described in He et al. [16], with some of the arrays improved (as noted in Table S1).

### 2.4. Metagenome Analysis

DNA preparation and PCR amplification of microbial DNA from the apple phyllosphere: All 27 leaves collected as above from one orchard were pooled, crushed into small pieces in liquid nitrogen, mixed well, and three aliquots (i.e., triplicates) of 1.0 g of leaf homogenate were taken for DNA extraction. To eliminate host chloroplast and mitochondrial DNA from the preparation, DNA extraction was performed as described in He et al. [16] according to Ikeda et al. [17] for enriching bacterial cells from plant organelles. Briefly, leaves were homogenized in bromochloropropane (BCP) buffer (5 mL for 1 g leaf), centrifuged at 5000× *g* for 1 min, and the supernatant collected and centrifuged at 5000× *g* for 1 min. The precipitate was dissolved in 1 mL BCP buffer, vigorously shaken for one second, and reprecipitated by centrifugation at 5000× *g* for 1 min. BCP buffer (1 mL) treatment was repeated, and the final precipitate was subjected to DNA extraction by the ISOPLANT II DNA extraction kit (Nippon Gene, Osaka, Japan).

DNA extraction was repeated three times from one sample, resulting in a total of nine DNA preparations, each 100 μL, from each orchard. The three DNA preparations from one sample were mixed well, and an aliquot of DNA (100 ng in 2 μL) was used for PCR amplification of fungal rDNA-ITS and of bacterial 16S rDNA region, with the primer set shown in Table S2. PCR was performed in a 25-μL mixture containing 2 μL of total DNA extract, 2.5 μL of 2.5 mM dNTPs, 2.5 μL of 10× LA PCR buffer, 2.5 μL of 25 mM MgCl_2_, 0.25 μL of LA-Taq DNA polymerase (Takara Bio, Shiga, Japan). Cycle parameters for PCR were heat-denaturation at 94 °C for 4 min, followed by 30 cycles of amplification (94 °C for 1 min; 55 °C for 1 min; 72 °C for 1 min), and a final extension at 72 °C for 7 min. The amplified DNAs were extracted twice by equal volumes of phenol:chloroform (1:1), precipitated by ethanol and dissolved in 50 μL of distilled water. The PCR amplification was repeated three times for each DNA mixture, and finally, nine PCR products were pooled for sequencing as a representative of one orchard. The pooled samples were electrophoresed in 7.5% polyacrylamide gels, after which the DNA amplicons (ranging in size from 300 to 700 bp) were recovered and sent to Hokkaido System Science (Sapporo, Japan) for large-scale sequence analysis by 454 GS Junior sequencer (Titanium, Roche) using reagents/protocols supplied by the manufacturer. Genus or species were identified by BLASTn analysis of the sequences obtained against the rRNA/ITS databases of NCBI and DDBJ. Those showing sequence homology higher than 98% were identified as species. Sequence homology was calculated using the pairwise nucleotide sequence alignment algorithm and displayed automatically on the BLASTn screen in the databases.

DNA preparation and PCR amplification of microbial DNA from apple rhizosphere: Soil samples were collected from two orchards (Natural-AK and Chemical-O) on 30 July 2010, in essentially the same manner as Matsushita et al. (2019) [17]. Briefly, after passing through a filter (⌀ = 2 mm), samples were stored until use in a freezer at −80℃ on 6 August. Soil DNA was extracted from the frozen soil (0.5 g) by using ISOIL for Beads Beating reagent (Nippon Gene Co., Ltd., Toyama, Japan) according to the manufacturer’s instructions. Three replicates of DNA preparations per soil sample were prepared and used for PCR amplification of fungal rDNA-ITS with the primer set ITS1 (5′-TCCGTAGGTGAACCTGCGG-3′) and ITS4 (5′-TCCTCCGCTTATTGATATGC-3′) [18], and of bacterial 16S rDNA region with the primer set Bac16S-27F (5′-AGAGTTTGATCCTGGCTCAG-3′) and Bac16S-519R (5′- GWATTACCGCGGCKGCTG -3′). PCR amplification was performed three times for each DNA extract, for a total of nine PCR preparations per soil sample. These were then pooled and electrophoresed in 7.5% polyacrylamide gels, and DNA amplicons ranging in size from 300 to 700 bp were recovered for large-scale sequencing by Hokkaido System Science (as above). Nucleotide sequence data reported are available in the DDBJ Sequenced Read Archive under the accession numbers DRX300681-DRX300700.

### 2.5. Isolation of Pseudomonas Species

Leaves were collected for macroarray analysis as described previously [16]. Namely, all 27 leaves from three trees per one apple orchard were immersed in 100 mL of extraction buffer (0.85% NaCl, 0.01% Tween 20) and shaken vigorously for 1 h at 4 °C. The aqueous phase was serially diluted (10×, 100×, 500×) and plated 100 μL on an agar plate (φ = 9 cm) containing *Pseudomonas* agar base (Oxoid, code CM0559, Thermo Fisher Scientific, MA, USA) containing *Pseudomonas* C-F-C supplement (Oxoid, Thermo Fisher Scientific, MA, USA). Plates were incubated at 25 °C for 48 h. From hundreds or more colonies, 50 each with white and smooth, yellowish and smooth, and yellowish and rough were isolated. Then 10 each were randomly selected for PCR amplification and sequencing of bacterial 16S rDNA region by PCR using a primer set Bac-27F (5′-AGAGTTTGATCMTGGCTCAG-3′) and Bac-1379M (5′-TGATYYRCGATTACTAGCRAYTCC-3′). Obtained nucleotide sequences were trimmed and added a gap to make them the same length according to the output of ClustalW (https://clustalw.ddbj.nig.ac.jp/, accessed on 23 September 2021). The 16S rDNA sequences of five *Pseudomonas* sequences, *P. graminis* strain 4-2T (MK078275), *P. syringae pv. syringae* strain SHPS007 (KP753380), *P. fluorescens* strain 36F3 (KT695822), *P. putida* strain ZJUTBX04 (JF682514), and *P. oryzihabitans* strain YQHJ48 (MN327658) were used as references. The phylogenetic analysis was performed with an unweighted pair group method with arithmetic mean (UPGMA) with bootstrap values based on 1000 replications using the MEGA11 computer program (https://www.megasoftware.net/, accessed on 23 September 2021). Nucleotide sequences were deposited in NCBI under the GenBank accession numbers OK256071-256080 and OK256133-OK256152.

### 2.6. Statistical Analyses

The Simpson’s reciprocal index (1/D) was calculated by 1/D = $\frac{N\left(N-1\right)}{\sum n\left(n-1\right)}$, where *N* and *n* represent the total number of organisms founded and the number of individuals of a particular species, respectively. The Simpson’s index of diversity (1 − D) was calculated by (1 − D) $=1-\frac{\sum^{\;}n\left(n-1\right)}{N\left(N-1\right)}$. The Shannon–Wiener index (*H*′) was calculated by *H*′ = $-\sum^{\;}p_{i}lnp_{i}$, where *p*_i_ represents the proportion of sequences that belong to the ith species. Pielou’s evenness index (J′) was calculated by J′ = $\frac{H^{\prime }}{lnS}$, where *S* represents the number of species of interest.

## 3. Results

### 3.1. Disease Incidence and Microbial Diversity in the Phyllosphere of Natural and Conventional Apple Orchards

**Figure 1:**
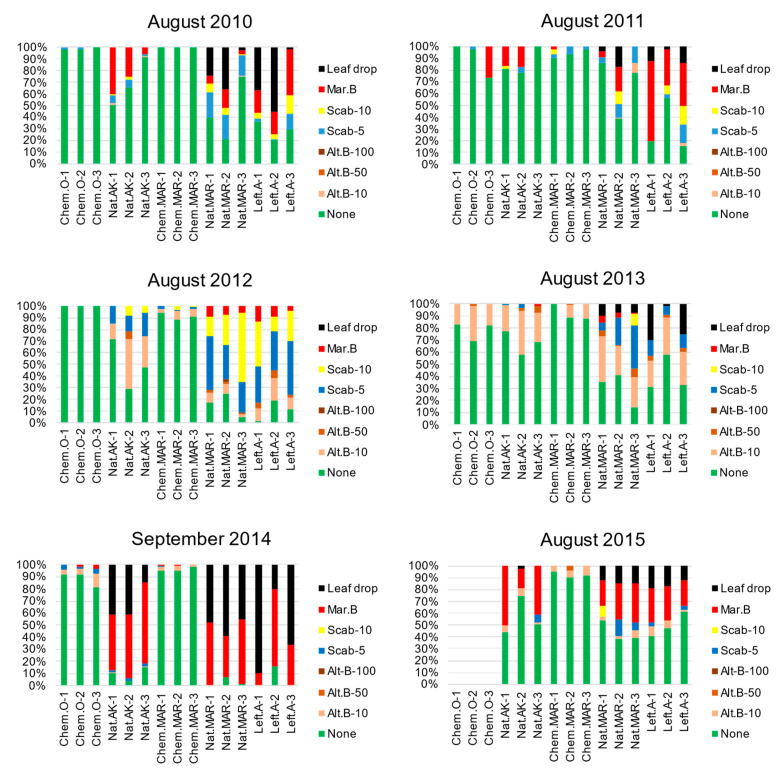
Disease incidence in orchards in August 2010–2015 seasons. Representative data showing the severity of leaf disease symptoms for scab, Alternaria blotch, and Marssonina blotch (and for early leaf drop caused by Alternaria blotch and Marssonina blotch), scored by counting 300 leaves per tree in the three selected trees. Chem-O, Nat-AK, Chem-MAR, Nat-MAR, and Left-A stand for Chemical-O, Natural-AK, Chemical-MAR, Natural-MAR, and Left-alone-MAR, respectively, with individual tree numbers indicated as 1–3. Alt-B-10, Alt-B-50, Alt-B-100 indicate the proportion of leaves with 1–10, 11–50, or 51–100 lesions of Alternaria blotch, respectively. Similarly, Scab-5 and Scab-10 are the proportion of leaves with 1–5 or 6–10 scab lesions, respectively. Mar.B is the proportion of leaves showing symptoms of Marssonina blotch. In 2014, data were collected in September because the survey for August could not be conducted. Samples from Chemical-O in 2015 were not included in the data set because the orchard could not be surveyed.

Overview of disease incidence in the 2010 to 2015 growing seasons: Monthly visual inspections of disease incidence from May to October in the 2010–2015 growing seasons revealed no serious epidemics in the conventionally farmed orchards (Chemical-O and Chemical-MAR); only minor scab and/or Marssonina blotch symptoms could be seen sporadically in September to October (the harvest season) after chemical pesticide spraying had finished. In contrast, epidemics of major apple diseases such as scab, Alternaria blotch, and Marssonina blotch were quite common in the natural farming orchards (Natural-AK and Natural-MAR) and the left-alone orchard (Left-alone-MAR). Monilinia blight was also prevalent in Natural-AK in early May (blooming time). To assess the differences, especially in the natural farming orchards, the severity of the symptoms of the aforementioned leaf diseases (including early leaf drop caused by Alternaria and Marssonina blotch) were scored. As measured in August, the severity of scab, Alternaria blotch, Marssonina blotch, and premature defoliation (caused mainly by Alternaria blotch and Marssonina blotch) were markedly less in Natural-AK (Nat-AK-1, -AK-2, -AK-3) compared to Natural-MAR and Left-alone-MAR throughout the years of observation (Figure 1).

### 3.2. Microbial Diversity in the Apple Phyllosphere by Macroarray Analysis

Monitoring by macroarray analysis of the diversity and the seasonal changes (June to October) of fungal and bacterial species in the apple phyllosphere in the 2010–2012 seasons revealed that abundance of microbial inhabitants, especially bacterial species, was rather limited in June to July but significantly increased from August to September (Supplementary Figure S1). Therefore, our analysis was focused primarily on the time period from August to September.

**Figure 2:**
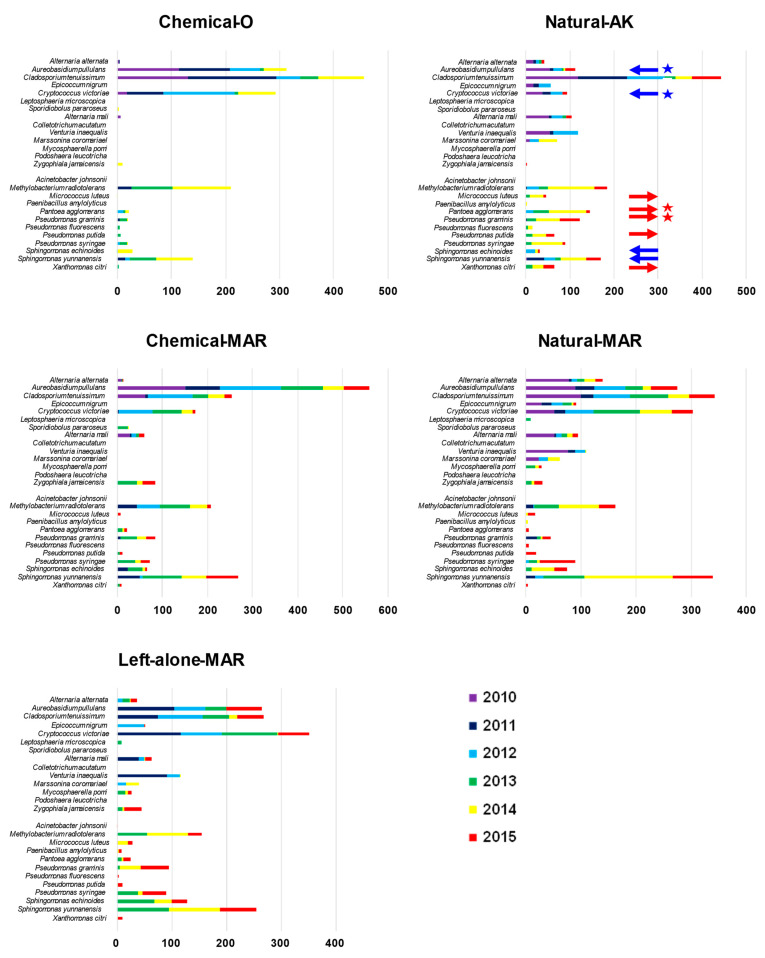
Differences in major fungal and bacterial species in the apple phyllosphere in August 2010–2015, quantified by intensity of hybridization signals obtained by macroarray analysis. Purple, blue, light blue, green, yellow, and red horizontal bars indicate the relative amounts of each fungus and bacterium in samples from 2010, 2011, 2012, 2013, 2014, and 2015, respectively. Red arrows indicate species more abundant in Natural-AK, blue arrows indicate species less abundant in Natural-AK. Samples from Chemical-O in 2015 were not included because the orchard could not be surveyed in 2015.

Fungi: Macroarray analysis of the diversity of fungal inhabitants in the apple phyllosphere conducted in the 2010–2015 seasons revealed that *Aureobasidium pullulans*, *Cladosporium tenuissimum*, and *Cryptococcus victoriae* were the predominant non-phytopathogenic species in both the natural and conventional farming orchards (Supplementary Figure S1, Figure 2). In addition, the phytopathogenic fungi *Alternaria. mali* (the causal pathogen of Alternaria blotch), *Venturia inaequalis* (scab), and *Diplocarpon mali* (Marssonina blotch) were prevalent in the two natural farming and the left-alone orchards. *V. inaequalis* and *A. mali* appeared from May to June, and both remained detectable throughout the growing season. *D. mali* normally appeared later, from August to October, giving rise to serious leaf damage and causing early leaf drop from September, resulting in a reduction in fruit quality.

We found considerably lower levels (relative ratios) of *A. pullulans* and *C. victoriae* in Natural-AK compared to the other orchards, including the two conventional orchards (Figure 2, blue arrows with star). For example, the relative ratio of *A. pullulans* in Natural-AK (total, June–October) was 8.7% in 2010, 2.2% in 2011, and 9.1% in 2012, lower than in Natural-MAR (19.3% in 2010, 23.4% in 2011, and 25.7% in 2012), Chemical-O (37.0% in 2010, 26.5% in 2011, and 27.5% in 2012) and Chemical-MAR (62.0% in 2010, 58.0% in 2011, and 37.7% in 2012), although these differences were statistically significant only between the Natural and chemical orchards. Rather than these two fungi, *C. tenuissimum* predominated in Natural-AK, with relative ratios of 41.8% in 2010, 74.9% in 2011, and 36.1% in 2012, higher than those in the other orchards. By contrast, no species predominated in Natural-MAR, where the relative ratios of the top five species ranged from 24.9% (1st) to 8.4% (5th) in 2010, 27.0% to 13.0% in 2011, and 25.9% to 5.7% in 2012, suggesting that the major fungal species co-existed more in the phyllosphere (Supplementary Figure S1).

Bacteria: *Methylobacterium radiotolerans* and *Sphingomonas yunnanensis* were the predominant species, both in natural farming and conventional orchards. As expected, the spectrum of bacterial species was generally wider and more balanced in Natural-AK, with none predominating to the extent found in the other orchards. *Pseudomonas* (including *P. graminis*, *P. syringae*, *P. putida,* and *P. fluorescens*) and *Pantoea agglomerans* were detected most frequently in Natural-AK (Figure 2, red arrows), while *S. yunnanensis* and *S. echinoides* were detected less in Natural-AK (Figure 2, blue arrows).

### 3.3. Metagenome Analysis by Next-Generation Sequencing

#### 3.3.1. Large-Scale Sequencing Analysis of Microbial Inhabitants in the Apple Phyllosphere

**Figure 3:**
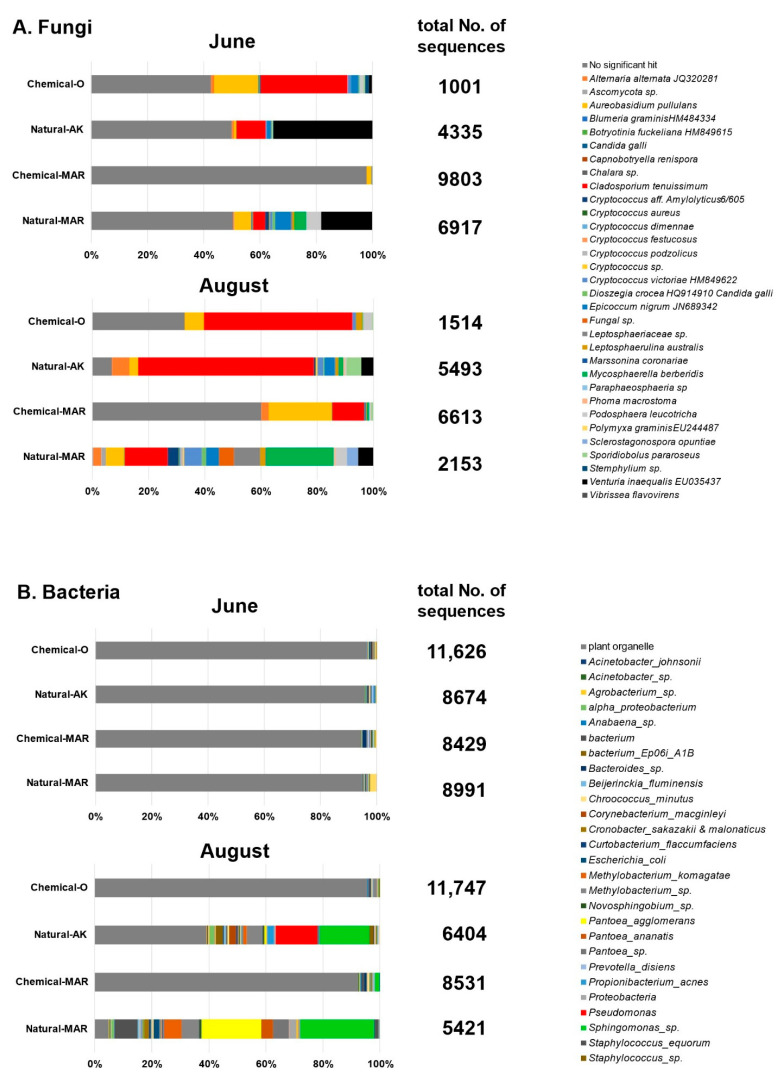
Proportions of (**A**) fungal and (**B**) bacterial species detected by next-generation sequencing from the apple phyllosphere in June and August 2010. In (**A**), “no significant hit” (gray) indicates hits of plant, not fungal, origin. In (**B**), “plant organelle” (gray) indicates hits for organelles (such as mitochondria) of prokaryotic, not bacterial, origin.

Overall profiling: Based on macroarray analysis results, two time points (12 June and 6 August 2010) and four orchards (Natural-AK, Chemical-O, Natural-MAR, and Chemical-MAR) were selected for metagenome analysis by next-generation sequencing. Since Natural-MAR had been most severely affected by diseases and pests in 2010, the second year after conversion to natural farming, we anticipated that data from this year would provide a contrast with Natural-AK. Results for these two months were 1001–9803 and 5421–11,747 reads (average length 500 bp) per sample, respectively, obtained from eight fungal and eight bacterial preparations. However, these results included a considerable number of reads derived from plant genomes, plant organelles, and from unknown origins, especially for the fungal samples (Figure 3A, gray). Notably, all four bacterial preparation samples from 12 June, along with those from the conventional orchards (Chemical-O and Chemical-MAR) from 6 August, contained a majority (>95%) of reads originating from plant mitochondrial and chloroplast DNA (Figure 3B, gray). These results can be explained by the fact that the absolute amounts of fungi and bacteria were low in June, as shown in the macroarray analysis; therefore, gene sequences of plant origin were relatively increased. The numbers of genera, species, and sequence reads of fungi and bacteria determined by BLAST analysis are summarized in Table 1. These data were used for subsequent analyses, omitting “no significant hit” in fungi and “plant organelle” in bacteria.

Although the number of species of fungi detected in June and August was not much different among the four orchards, there was a wide range in the total number of reads from individual orchards, with only 200 in Chemical-MAR in June but 5108 in Natural-AK in August. This difference illustrates the abundance of fungal populations inhabiting the apple phyllosphere in the natural farming orchards in August, a result supported by the macroarray analysis.

With respect to bacteria, both diversity of species and abundance (number of sequence reads) exhibited strong contrasts among the samples. The total number of reads for true bacteria was small in June. This result could be attributed to the dearth of bacteria in these samples, resulting in the extremely high relative ratio of prokaryote-type organelle DNA. By contrast, the number of reads for true bacteria, and species, increased dramatically in the August samples from the two natural farming orchards: 3887 reads consisting of 112 species from 107 genera for Natural-AK, and 5158 reads consisting of 102 species from 94 genera for Natural-MAR (Table 1).

**Figure 4:**
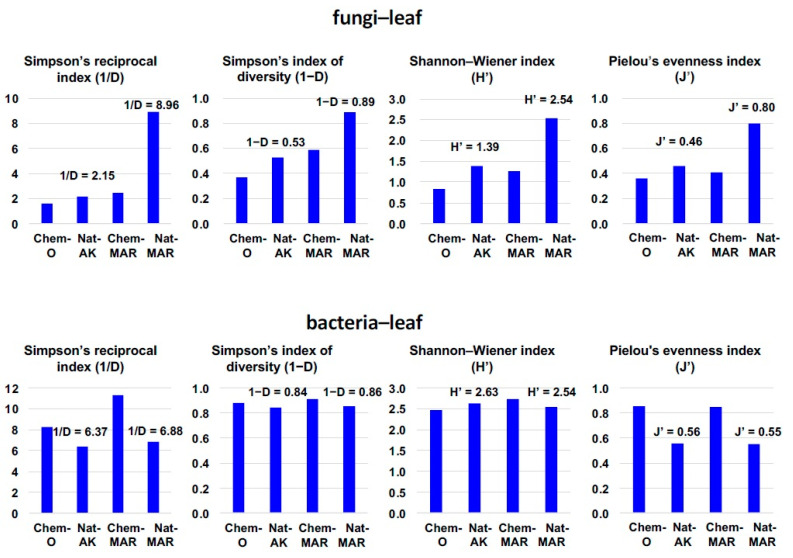
Statistical analysis of diversity and evenness of the top 18 fungi and top 15 bacteria inhabiting the apple phyllosphere in August 2010 detected by next-generation sequencing. Chem-O, Nat-AK, Chem-MAR, and Nat-MAR stand for Chemical-O, Natural-AK, Chemical-MAR, and Natural-MAR, respectively. Numbers above bars for Nat-AK and Nat-MAR show the index values.

Following the results on the abundance of fungi and bacteria inhabiting the apple phyllosphere in the natural farming orchards in August, their diversity was examined statistically using Simpson’s index of diversity, Simpson’s reciprocal index, the Shannon-Weiner index, and Pielou’s evenness index. All four indices indicated that the diversity of fungi was highest in Natural-MAR and that diversity in Natural-AK was not much different from Chemical-O or Chemical-MAR. On the other hand, these indices indicated that bacterial diversity was almost the same among the four orchards, although using Simpson’s reciprocal and Pielou’s evenness indices, bacterial diversity tended to be higher in the two conventional orchards, Chemical-O and Chemical-MAR (Figure 4). These results, when taken together with the severity of disease incidents in Natural-AK and Natural-MAR, indicate that microbial diversity in the phyllosphere is not the key determinant for the suppression of disease observed in Natural-AK. Rather, it should be noted that there were no dominant fungal species in Natural-MAR, whereas one or two fungal species (*C. tenuissimum* and *A. pullulans*) dominated in Natural-AK, as well as in Chemical-O and Chemical-MAR.

Profiling of fungal diversity: All sequencing results contained a large proportion of reads with “no significant hit.” The proportions of hits of fungal origin in June were 49.9% in Natural-AK, 49.6% in Natural-MAR, 57.3% in Chemical-O, and 2.0% in Chemical-MAR, which increased to 93.0%, 99.7%, 67.2%, and 39.8%, respectively, in August (Table 1). Major fungal species identified were consistent with those identified by macroarray analysis: phytopathogenic—*V. inaequalis*, *A. alternata* or *A. mali* and *D. mali*; non-phytopathogenic—*C. tenuissimum*, *E. nigrum*, *A pullulans*, *Cryptococcus* spp. and *Sporidiobolus pararoseus*. In addition, the significant presence of *M. pomi* (causal pathogen of Brooks fruit spot) and *Podosphaera leucotricha* (powdery mildew) were detected in the natural farming orchards.

**Figure 5:**
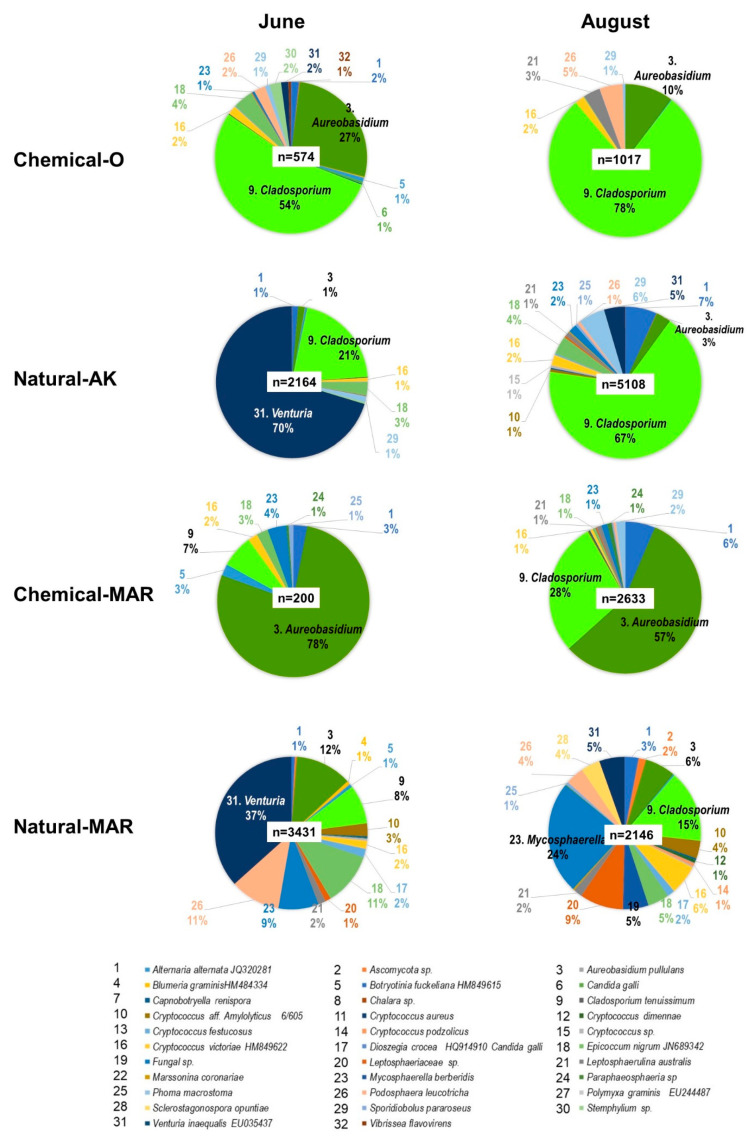
Analysis by next-generation sequencing of fungal species diversity in the apple phyllosphere in four orchards. Samples prepared from leaves collected in June (upper) and August (lower) were analyzed by 454 GS Junior (Titanium, Roche). A total of 32 species were identified, as listed. Those constituting less than 1% are not shown in the pie charts. “n” is the total number of reads identified as fungi. Note the considerably lower “n” values in samples from Chem-O and Chem-MAR in June.

Among the phytopathogenic fungi in Natural-AK and Natural-MAR, *V. inaequalis* was by far the most dominant species in June (70.3% and 36.6% of total reads, respectively), although by August it comprised only 4.5% and 5.4% of the total, respectively (Figure 5). Ratios for the other phytopathogenic fungi increased from June to August, but the rate of increase was moderate in Natural-AK: from 1.3% to 6.8% for *Alternaria* spp., from 0.0% to 1.0% for *P. leucotricha*. Among the non-phytopathogenic species, *C. tenuissimum* dominated in June (20.6%) and increased further in August (67.2%). *E. nigrum*, *C. victoriae*, *S. pararoseus*, and *A. pullulans* were second most dominant, increasing from June to August slightly: 2.8–3.9%, 0.8–2.3%, 1.1–5.6%, and 1.3–3.2%, respectively. In Natural-MAR, *P. leucotricha* ranked second highest among the phytopathogenic species in June (10.6%), maintaining a relatively high ratio in August (3.9%), although powdery mildew was not prevalent in the orchard. *M. pomi* (or *M. berberidis*) increased from 8.6% in June to become the most predominant in August (24.0%), resulting in the emergence of black spot disease. *Alternaria* spp. was present at low levels: 0.8% and 3.0% in June and August, respectively. Among non-phytopathogenic species, the ratio of *C. tenuissimum* was not as high as in Natural-AK, ranging from 8% to 15.3% in June and August, and comparable to those of *A. pullulans* (12.0–6.4%), *E. nigrum* (11.2–4.5%), and *C. victoriae* (2.0–6.3%).

In the conventional orchards, the phytopathogenic species were detectable, but the numbers of reads were limited. Among the non-phytopathogenic species, *A. pullulans* and *C. tenuissimum* were the hyper dominant species in both chemical orchards. In Chemical-O, the proportion of *C. tenuissimum* was 53.8% in June, increasing to 78.4% in August, and for *A. pullulans,* 27.2% in June and 10.1% in August. In Chemical-MAR, *A. pullulans* dropped from 77.5% in June to 56.8% in August, while *C. tenuissimum* rose from 7.0% in June to 28.4% in August. The top two species in these conventional orchards occupied more than 80% of the population in both months.

Profiling of bacterial diversity: In June, plant organelle reads constituted more than 99% of the total, indicating that the relative proportion of bacteria was very low in the phyllosphere (Figure 3B). In the conventional orchards (Chemical-O and Chemical-MAR), the proportion of bacterial origin remained very low (<10% or 537–661 counts), and species diversity was very limited, even in August, suggesting that both the abundance and diversity of bacterial inhabitants in the phyllosphere were severely affected by chemical pesticides. In contrast, the proportion of reads of bacterial origin rose to 60.7% (3887 counts) in Natural-AK and to 95.1% (5158 counts) in Natural-MAR in August, indicating a greatly increased abundance and diversity of bacteria in the natural farming orchards in August (when weather conditions were hot and humid). The genera *Sphingomonas*, *Methylobacterium*, *Pseudomonas*, and *Acinetobacter*, along with *Bacteroides* sp. and *P. agglomerans*, were among those commonly detected from every sample, a result in agreement with the macroarray data.

**Figure 6:**
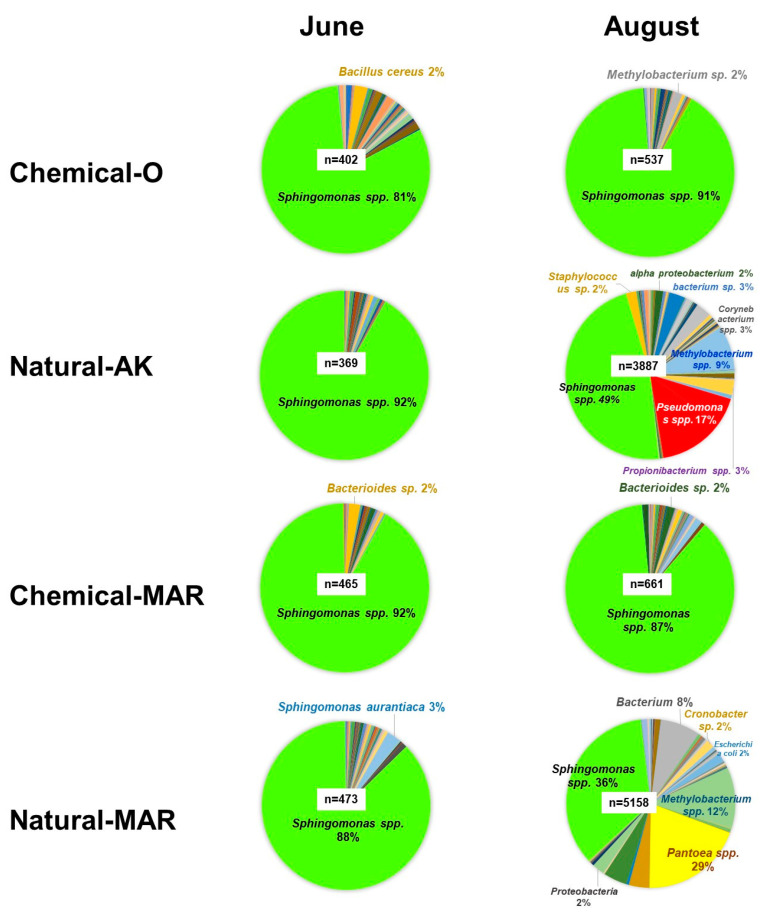
Analysis by next-generation sequencing of bacterial species diversity in the apple phyllosphere in four orchards. Samples prepared from leaves collected in June (upper) and August (lower) were analyzed by 454 GS Junior (Titanium, Roche). A total of 20 species were identified, and those with a proportion higher than 2% are shown in the pie graphs. “n” is the total number of reads identified as bacteria. Light green indicates *Sphingomonas* spp., red indicates *Pseudomonas* spp., yellow indicates *Pantoea* spp., and light blue indicates *Methylobacterium* spp.

In June, although the number of reads of bacterial origin was limited (369–473 counts), the predominant genera/species were: *Sphingomonas* (92%), *Pseudomonas* (1.1%), *Bacteroides* (0.8%), and *Propionibacterium* (0.8%) in Natural-AK; *Sphingomonas* (81%), *Bacillus* (5.7%, including *B. cereus*, 2%), *Bacteroides* (1.5%), and *Pseudomonas* (0.7%) in Chemical-O; *Sphingomonas* (88%) and *Pseudomonas* (1.3%) in Natural-MAR; and *Sphingomonas (92%)*, *Bacteroides* (2.2%), *Novosphingobium* (0.9%), and *Pseudomonas* (0.4%) in Chemical-MAR (Figure 6).

In August, *Sphingomonas* still predominated in all the orchards. In Chemical-O, *Bacillus* was not detected, and *Methylobacterium* increased, while in Chemical-MAR, *Novosphingobium* was not detected. In Natural-AK and Natural-MAR, in addition to *Sphingomonas*, *Methylobacterium* was relatively abundant (9–12%). While *Pseudomonas* species were detected in all the samples in June, it was observed that their ratio increased extensively only in Natural-AK (to 17% of the total read of bacterial origin). Meanwhile, *P. agglomerans* increased to 29% in Natural-MAR, becoming the predominant species there (Figure 6).

#### 3.3.2. Large-Scale Sequencing Analysis of Microbial Inhabitants in the Apple Rhizosphere

Metagenome analysis was performed using soil DNA collected in 2010 from the rhizosphere in Natural-AK and Chemical-O. Samples from Natural-MAR and Chemical-MAR were not used for this analysis because the location and the quality of the soil in these orchards were too different from that of Natural-AK. A total of 4500–20,300 reads (average length of 500 nucleotides) were obtained from fungal and bacterial DNA samples prepared from Natural-AK and Chemical-O. Among these, a total of 2351 reads for sequences of fungal origin and 9628 reads of bacterial origin were identified from Natural-AK, and 5329 and 11,727 reads for sequences of fungal and bacterial origin, respectively, were identified from Chemical-O.

Profiling of fungal diversity: With respect to fungi, surprisingly, 92.1% of reads from Natural-AK and 89.4% from Chemical-O did not show any significant hits for known taxonomic genera; the remainder included 50 and 55 genera from Natural-AK and Chemical-O, respectively. The order of abundance was *Emericella*, *Fusarium*, *Mortierella*, *Cordyceps*, and *Leohumicola* in Natural-AK, and *Fusarium*, *Cryptococcus*, *Verticillium*, *Dipodascus*, and *Leohumicola* in Chemical-O (Table 2, Supplementary Figure S2). The genus *Fusarium* (47%) predominated in Chemical-O, and the top five genera encompassed nearly 75% of the population. In Natural-AK, the top five genera encompassed nearly 50%, and the top 15 encompassed 75% of the population, suggesting that fungal diversity was higher in Natural-AK than Chemical-O. It should be noted that *Cordyceps* and *Arthrobotrys*, genera known to include insect- or nematode-infecting species, were found only in Natural-AK (0.34% and 0.13%, respectively), probably reflecting higher biodiversity in the orchard.

Profiling of bacterial diversity: A majority of sequences from bacteria (65.9% from Natural-AK and 73.5% from Chemical-O) showed no significant hits for any known taxonomic genera; the remainder from Natural-AK (3287 reads) and Chemical-O (3104 reads) included 327 and 356 genera, respectively. The order of abundance of genera was *Nitrospira*, *Bradyrhizobium*, *Cupriavidus*, *Burkholderia*, *Pseudomonas*, *Methylibium*, and *Flavobacterium* in Natural-AK, and *Nitrospira*, *Bradyrhizobium*, *Ktedonobacter*, *Bacillus*, *Methylosinus*, *Thermosporothrix*, and *Methylocystis* in Chemical-O (Table 2, Supplementary Figure S3). The top 15 in Natural-AK and the top 20 in Chemical-O occupied ca. 50% of each of the populations. Although the genus *Nitrospira* was most abundant in both orchards, its level in Natural-AK was more than double that found in Chemical-O. Considering the general importance of *Nitrospira* for nitrogen oxidation, the abundance of *Nitrospira* in Natural-AK is well in accordance with the abundance of undergrowth weeds in Natural-AK, which are allowed to grow vigorously before being cut twice a year and left onsite as green manure. In contrast, weeds are cut frequently and killed by herbicides in Chemical-O, with chemical fertilizers and manures added to supplement nutrition. The second most dominant bacterial genus, *Bradyrhizobium*, a legume-symbiotic nitrogen-fixing genus, was also quite abundant in Natural-AK.

### 3.4. Pseudomonas in Natural-AK

**Figure 7:**
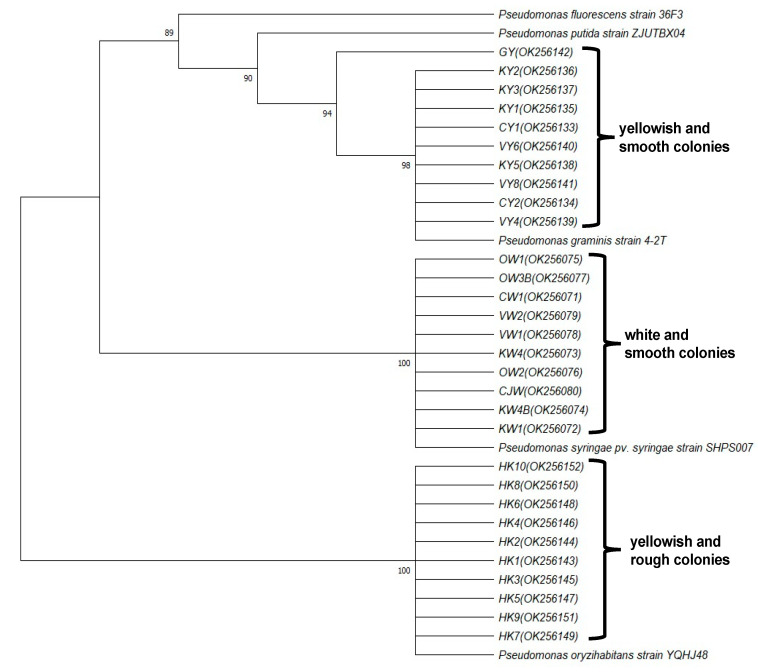
Phylogenetic analysis of 30 *Pseudomonas* colonies based on partial sequence of 16S rDNA. The dendrogram was generated by UPGMA. *P. graminis* strain 4-2T*, P. syringae pv. syringae* strain SHPS007, *P. fluorescens* strain 36F3, *P. putida* strain ZJUTBX04, and *P. oryzihabitans* strain YQHJ48 were used as references. Bootstrap percentage of 85% or more is indicated at the branch points. CW1, KW1, KW4, KW4B, OW1, OW2, OW3B, VW1, VW2, and CJW represent white and smooth colonies, CY1, CY2, KY1, KY2, KY3, KY5, VY4, VY6, VY8, and GY denotes yellowish and smooth colonies, and HK1–HK10 indicates yellowish and rough colonies, respectively. GenBank accession numbers of *Pseudomonas* colonies sequenced in the present study are shown in the parenthesis.

**Figure 8:**
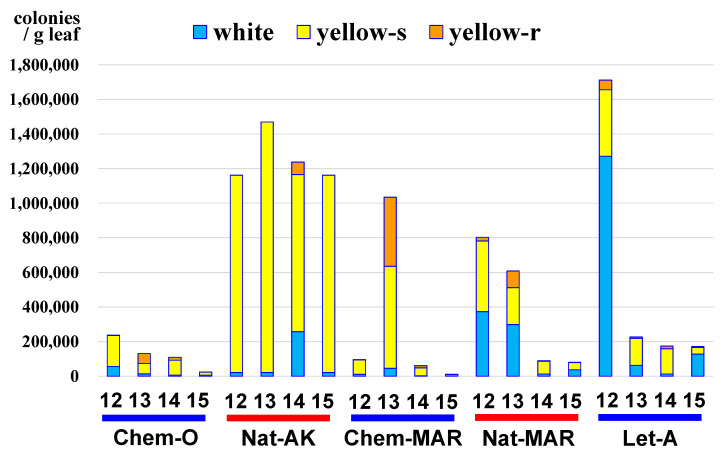
Numbers of *Pseudomonas* colonies (per gram leaf) isolated from apple phyllosphere using *Pseudomonas*-selective media from August 2012 (12 in the figure) to 2015 (15 in the figure). Samples were collected on August 9–11 in 2012, 2013, and 2015, and on September 4 in 2014. White, *Pseudomonas* with white colony; yellow-s, yellowish smooth-surface colony; yellow-r, yellowish rough-surface colony. *P. syringae* produces white and smooth colonies, *P. graminis* produces yellowish and smooth-surface colonies, and *P. oryzihanitants* produces yellowish and rough-surface colonies. Chem-O, Nat-AK, Chem-MAR, Nat-MAR, and Let-A represent Chemical-O, Natural-AK, Chemical-MAR, Natural-MAR, and Let-alone-MAR, respectively.

Since some *Pseudomonas* species were identified as major bacterial inhabitants in the apple phyllosphere by metagenome analysis in the August 2010 sample, the abundance and seasonal changes of the *Pseudomonas* population were analyzed during the 2012–2015 seasons in culture using *Pseudomonas*-selective media. Two main species of *Pseudomonas* with different colony colors and types were detected among all the orchards, with one white and smooth and one yellowish and smooth. In addition, a third species, yellowish and rough, was detected from some orchards. Nucleotide sequencing of 16S rDNA followed by phylogenetic analysis identified the white and smooth species as *P. syringae*, the yellowish and smooth as *P. graminis*, and the yellowish and rough as *P. oryzihabitants* (Figure 7). Most interestingly, *P. graminis* markedly predominated in Natural-AK compared to the other orchards, being especially abundant and hyper dominant in August 2012–2015 (Figure 8). This result was reproducible and consistent with the metagenome analysis from August 2010 (Figure 6).

## 4. Discussion

In our analysis of Natural-AK, the apple orchard naturally farmed for more than 30 years, we found that a series of major apple diseases (Monilinia blight in early spring, apple scab (*V. inaequalis*) in June–July, Alternaria blotch in July–August and Marssonina blotch in late August/the harvest season) prevailed considerably in every year throughout the growing seasons observed. On the major cultivar “*Fuji*”, for example, symptoms of Marssonina blotch appeared on 90–100% of leaves and caused ca. 10–20% premature defoliation by early October. The disease incidence was clearly worse than in the neighboring conventional orchards, including Chemical-O. However, we also found that disease was less severe in Natural-AK than in Natural-MAR (an orchard created to mimic Natural-AK) over the six-year period: in Natural-MAR, all the leaves developed severe symptoms of Marssonina blotch, and 50–95% of the leaves had fallen on the same cultivar over the same period.

We analyzed changes in major phytopathogenic and non-phytopathogenic fungi and bacteria by DNA macroarray in parallel to the above observations regarding the severity of major diseases and found that in the apple phyllosphere in the conventional orchards, the most predominant non-phytopathogenic fungal species were *A. pullulans*, *C. tenuissimum*, and *C. victoriae*, which is consistent with the reports from New Zealand and Switzerland that *A. pullulans* and *C. tenuissimum* were among those frequently isolated from apple leaves [5,19]. While in Natural-AK, *A. pullulans* and *C. victoriae* were considerably less abundant, and only *C. tenuissimum* was predominant. Interestingly, the same tendency could be seen in conventionally farmed Chemical-O. The diversity of fungal species in the phyllosphere was higher in Natural-AK than in Chemical-O but was lower than in Natural-MAR. Richness in fungal diversity among the two naturally farmed orchards (Natural-AK and Natural-MAR) was attributed to the abundance of phytopathogenic fungal species (*V. inaequalis*, *A. mali*, and *D. mali*), suggesting that the richness of non-phytopathogenic fungal diversity in the phyllosphere is not the key factor for the disease suppression observed in Natural-AK. Rather, the stable maintenance of balanced fungal species under a certain predominant one such as *C. tenuissimum* in the phyllosphere may be the basis for the intermediate level of disease incidence observed in Natural-AK. Prolonged natural farming practices, including periodic spraying with edible vinegar solutions, might have contributed to maintaining an ordered microbial population in the phyllosphere.

With respect to the bacterial constitution of the phyllosphere, results showed that both species diversity and richness were higher in Natural-AK and Natural-MAR than in Chemical-O and Chemical-MAR. While analyses by macroarray and metagenome sequencing identified *M. radiotolerans* and *Sphingomonas* species as predominant in all orchards, the abundance of *Pseudomonas* species increased conspicuously in Natural-AK in August, which was more apparent in the results obtained by metagenome analysis. Further analysis conducted in 2012–2015 by conventional culture methods using *Pseudomonas*-selective media followed by nucleotide sequencing of small subunit ribosomal RNA revealed that *P. graminis* increased and hyper dominated in Natural-AK in August for four consecutive years. It is known that *Pseudomonas* species, mainly those inhabiting the rhizosphere, such as *P. fluorescens*, can induce systemic resistance responses in plants and thus suppress diseases [20,21,22]. In our supplementary trial using tobacco–TMV as a model, *P. graminis* isolated from apple leaves was observed to induce higher levels of pathogenesis-related protein 3 (ntPR3) mRNA and induced resistance in tobacco leaves to TMV infection (Supplementary data). Since *P. graminis* CPA-7, used as a biocontrol agent, was reported to trigger the activation of the fruit defense response, thereby mitigating its oxidative damage [23], the abundant and hyper dominant *P. graminis* found in Natural-AK early in August may also contribute to suppression of subsequent development of diseases in Natural-AK through the induction of defense responses. This intriguing possibility holds great promise for future research.

Metagenomic analysis of microbial diversity in the rhizosphere revealed that fungal diversity was higher in Natural-AK than in Chemical-O. It is interesting to note that *Cordyceps*, a genus of ascomycete fungi known as endoparasitoids (parasitic mainly on insects and other arthropods) [24], was more abundant in Natural-AK than in Chemical-O. In addition, *Arthrobotrys* spp., predatory fungi that capture and feed on nematode worms [25], were also observed in Natural-AK. Both of these genera seemed to be associated with a rich and abundant undergrowth of weed flora, cut only twice a year and left onsite as green manure in Natural-AK. Probably for a similar reason, an important bacterial genus found inhabiting the rhizosphere was *Nitrospira*. Although the diversity of bacteria in the rhizosphere was not markedly different between Natural-AK and Chemical-O, the relative abundance of *Nitrospira* spp., the predominant species among the soil bacteria in Natural-AK, was more than double that found in Chemical-O. Since *Nitrospira*, in general, is known to be abundant in soil and to perform nitrite oxidation [26], our findings indicate that the capacity for nitrogen metabolism in the soil of Natural-AK should be higher than that of Chemical-O.

As mentioned above, Natural-AK has been maintained for many years without the use of chemical fertilizers or manures. Instead, abundant undergrowth weeds are left onsite after cutting as green manure to circulate nitrogen in the orchard. This practice appears to have established a superior soil microbial system in Natural-AK that is able to decompose and metabolize the green manure efficiently. The genus *Bradyrhizobium* was the second most predominant bacterial species in Natural-AK and was more abundant there than in Chemical-O. These are Gram-negative soil bacteria that can form symbiotic relationships with leguminous, nitrogen-fixing plant species. In Natural-AK, there has been a history of cultivating leguminous species as undergrowth weeds to aid nitrogen fixation, which is thought to have encouraged the establishment of soil environmental conditions suited to inhabitation by *Bradyrihzobium* spp. These results on soil bacteria were consistent with our previous report that richness and relative abundance of particular operational taxonomic units belonging to *Nitrospirae* and *Rhizobiales,* including close relatives *Bradyrhizobium,* were significantly higher in Natural-AK than in Chemical-O [17]. Increased numbers of nitrogen oxidation and fixation bacteria seem to have contributed to the establishment of a system in which nitrogen circulates effectively through the orchard soil, enhancing the vitality of apple trees, in turn improving their ability to combat pathogens and pests attacking the above-ground parts, limiting damage.

**Figure 9:**
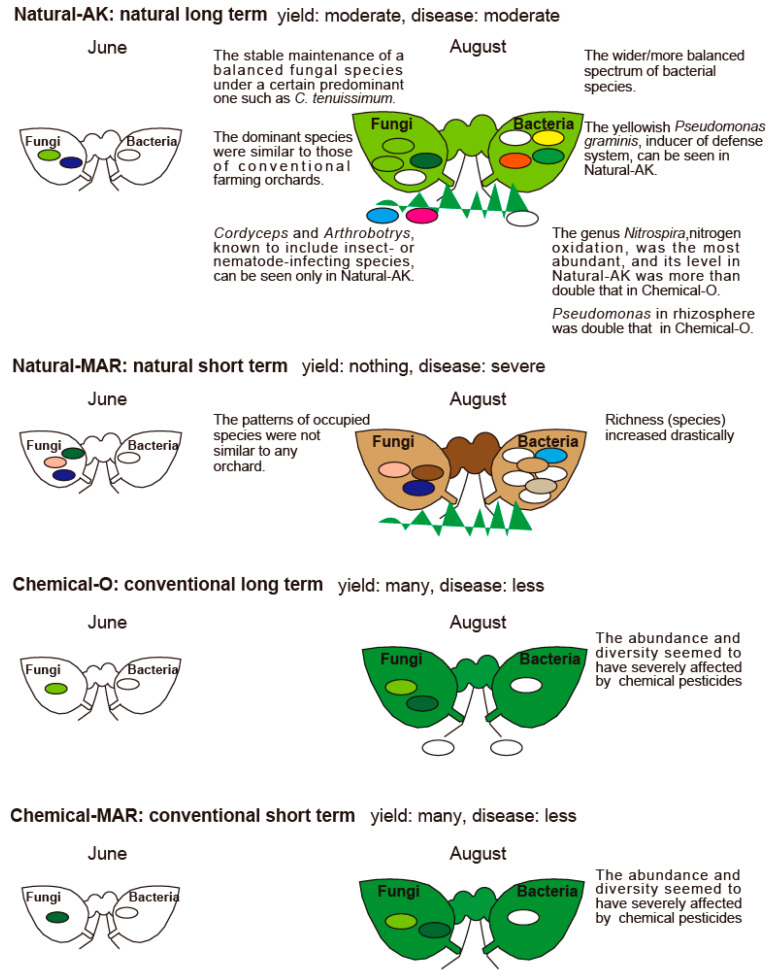
Summary of the 4 apple orchards in this study. The main points of yield, disease, fungal and bacterial characteristics are represented. Left and right sides of the tree represent the fungal and bacterial characteristics, respectively.

The current study comparing natural farming with conventional farming demonstrated the shift in microbial population during the growing season and cultural practice-dependent microbial population dynamics. Long-term natural farming favored the microbial population known to induce a plant’s defense system and be detrimental to insects. On the other hand, comparing short-term natural farming with long-term natural farming indicated that, longer time is required to re-establish the microflora when the orchard is converted from conventional farming to natural farming, which eventually helps to harvest suitable quality apples. As summarized in Figure 9, the present study focuses on the fungal and bacterial population in the phyllosphere and rhizosphere of apple orchards under different farming conditions. However, a more robust study including soil viral population, abiotic factors that directly and indirectly contribute to defining soil fertility, and plant health will help identify positive factors worthy of future application in apple cultivation. Additionally, the microbial characteristics observed in the present study need to be further investigated in other orchards and crops under similar farming practices to develop a microbial consortium for developing better soil management strategies. These developments should improve productivity by considering the environment and extending and augmenting previously established advanced farming techniques.

## Figures and Tables

**Table 1:** Numbers of genus, species, and sequences of fungi and bacteria detected in phyllosphere by next-generation sequencing.

	Fungi				Bacteria			
	No. Genus	No. Species	No. Fungal Sequence	Total No. of Read *	No. Genus	No. Species	No. Bacterial Sequence	Total No. of Reads *
June								
Chemical-O	18	20	574	1001	23	29	402	11,626
Natural-AK	18	20	2164	4335	18	19	369	8674
Chemical-MAR	9	10	200	9803	16	17	465	8429
Natural-MAR	15	21	3431	6917	25	28	473	8991
August								
Chemical-O	10	10	1017	1514	17	18	537	11,747
Natural-AK	18	22	5108	5493	107	112	3887	6404
Chemical-MAR	18	21	2633	6613	23	25	661	8531
Natural-MAR	18	25	2146	2153	94	102	5158	5421

* Total number of reads, including those of “no significant hit” in fungi and “plant organelle” in bacteria.

**Table 2:** Top 10 genus and occupancy from large-scale sequencing analysis of fungi and bacteria in the apple rhizosphere.

	*Genus*	Natural-AK	Chemi-O
		%	%
Major fungi	*Emericella*	**2.17**	0.19
	*Fusarium*	**0.94**	**4.95**
	*Mortierella*	**0.51**	0.23
	*Leohumicola*	**0.34**	**0.36**
	*Cordyceps*	**0.34**	0
	*Leptosphaeria*	**0.26**	0.02
	*Glomus*	**0.26**	0
	*Cryptococcus*	**0.21**	**1.5**
	*Zalerion*	**0.17**	**0.13**
	*Ophiosphaerella*	**0.17**	0
	*Cryptococcus*	0.21	**1.50**
	*Verticillium*	0.09	**0.62**
	*Dipodascus*	0	**0.43**
	*Sporidiobolus*	0	**0.23**
	*Pichia*	0	**0.19**
	*Cladosporium*	0	**0.13**
Major bacteria	*Nitrospira*	**6.12**	**2.12**
	*Bradyrhizobium*	**1.85**	**1.19**
	*Cupriavidus*	**1.13**	0.32
	*Burkholderia*	**1.00**	**0.76**
	*Pseudomonas*	**0.96**	0.47
	*Stigmatella*	**0.84**	0.16
	*Methylibium*	**0.80**	0.08
	*Flavobacterium*	**0.77**	0.26
	*Methylosinus*	**0.68**	**0.84**
	*Anaeromyxobacter*	**0.65**	0.26
	*Ktedonobacter*	0.45	**0.99**
	*Bacillus*	0.12	**0.96**
	*Thermosporothrix*	0.16	**0.82**
	*Methylocystis*	0.32	**0.77**
	*Legionella*	0.27	**0.76**
	*Candidatus Solibacter*	0.38	**0.68**

Top 10 genus found in Natural-AK and Chemi-O are shown by bold numbers.

## Data Availability

Nucleotide sequence data reported are available in the DDBJ Sequenced Read Archive under the accession numbers DRX300681-DRX300700 and in GenBank under the accession numbers OK256071-256080 and OK256133-OK256152.

## Supplementary Materials

The following are available online at https://www.mdpi.com/article/10.3390/microorganisms9102056/s1, Figure S1: Proportional changes in major fungal species in apple phyllosphere from June (JUN) to October (OCT) in 2010–2012 seasons. Figure S2: Analysis by next-generation sequencing of fungal species in the rhizosphere in two apple orchards, Chemical-O (Chem-O) and Natural-AK (Nat-AK). Figure S3: Analysis by next-generation sequencing of bacterial species in the rhizosphere in two apple orchards, Chemical-O (Chem-O) and Natural-AK (Nat-AK). Supplementary Data S1: Analysis of induced resistance by *Pseudomonas* using tobacco mosaic virus challenge infection assay. Table S1: List of arrays for macroarray analysis—names, nucleotide sequence, and accession number. Table S2: List of primers.
