# Supplementary material for: Microbial Diversity in the Phyllosphere and Rhizosphere of an Apple Orchard Managed under Prolonged “Natural Farming” Practices
Source: Microorganisms. 2021 Sep 29;9(10):2056. doi: 10.3390/microorganisms9102056 (PMC8540600; doi:10.3390/microorganisms9102056)
Supplement: Supplementary file 1 [file microorganisms-09-02056-s001.zip › Figure S1 (He et al.,).pdf]

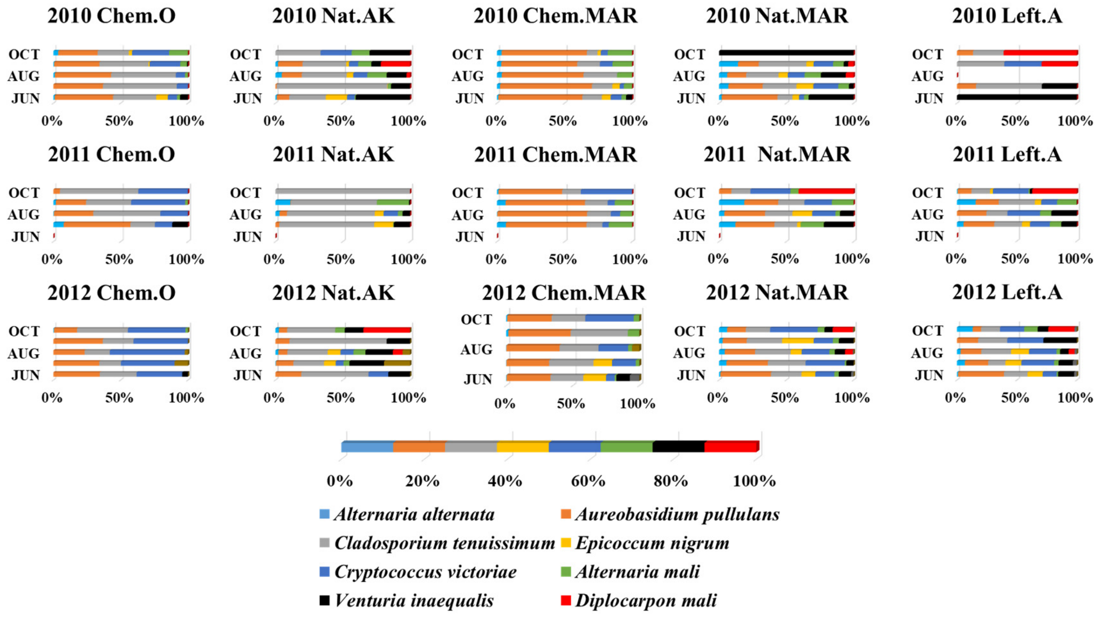

Figure S1. Proportional changes in major fungal species in apple phyllosphere from June (JUN) to October (OCT), 2010–2012.

Intensity of hybridization signal obtained by macroarray analysis of the major pathogenic and nonpathogenic fungi on the apple phyllosphere was quantified. Macroarray analysis was performed individually for three tree samples per orchard and averaged. Chem.O, Nat.AK, Chem.MAR, Nat.MAR, and Let.A stand for Chemical-O, Natural-AK, Chemical-MAR, Natural-MAR, and Let-alone-MAR, respectively. Red arrow indicates the relatively high abundance of *C. tenuissimum* in Natural-AK. Blue arrows indicate the relatively low abundances of *A. pullulans* and *C. victoriae* in Natural-AK.
