# Supplementary material for: Microbial Diversity in the Phyllosphere and Rhizosphere of an Apple Orchard Managed under Prolonged “Natural Farming” Practices
Source: Microorganisms. 2021 Sep 29;9(10):2056. doi: 10.3390/microorganisms9102056 (PMC8540600; doi:10.3390/microorganisms9102056)
Supplement: Supplementary file 1 [file microorganisms-09-02056-s001.zip › Figure S3 (He et al.,).pdf]

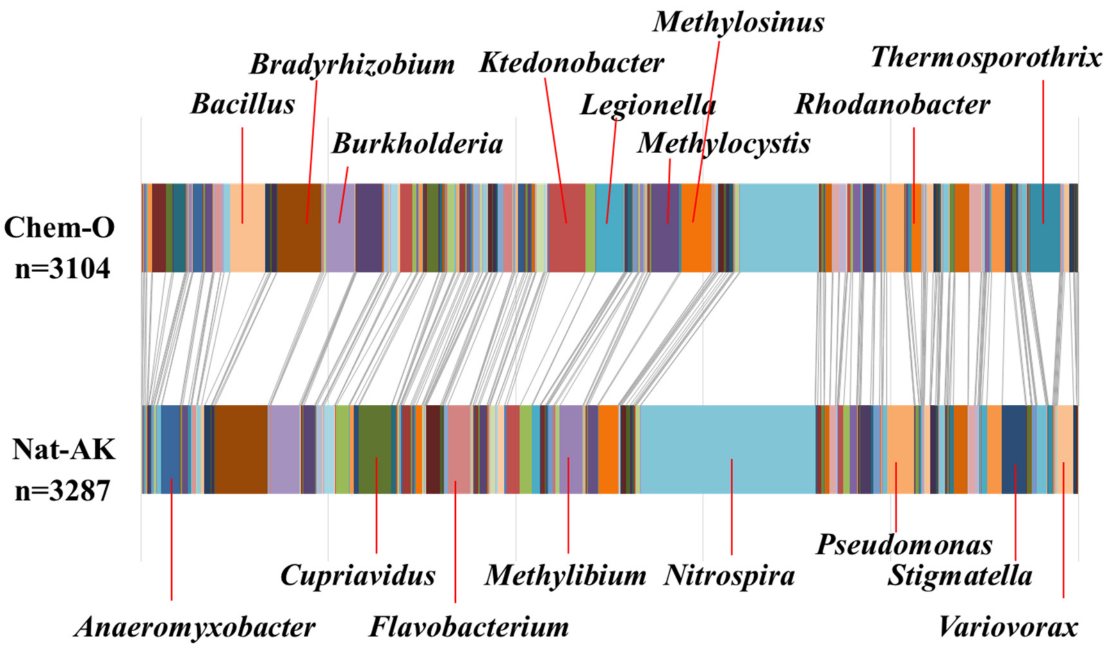

Figure S3. Analysis by next-generation sequencing of bacterial species in the rhizosphere of two apple orchards, Chemical-O (Chem-O) and Natural-AK (Nat-AK).

Samples prepared from soils collected in Aug 2010 were analyzed by 454 GS Junior sequencer (Titanium, Roche). Among the sequences obtained from Chemical-O (n = 11,727) and Natural-AK (n = 9,628), 3,104 and 3,287, respectively, hit known taxonomic genera (356 in Chemical-O and 327 in Natural-AK).
