# Supplementary material for: Microbial Diversity in the Phyllosphere and Rhizosphere of an Apple Orchard Managed under Prolonged “Natural Farming” Practices
Source: Microorganisms. 2021 Sep 29;9(10):2056. doi: 10.3390/microorganisms9102056 (PMC8540600; doi:10.3390/microorganisms9102056)
Supplement: Supplementary file 1 [file microorganisms-09-02056-s001.zip › Supplementary data 1 (He et al.,).pdf]

Supplementary Data S1. Analysis of induced resistance by *Pseudomonas* using tobacco mosaic virus challenge infection assay

Three species of *Pseudomonas* bacteria detected from apple orchards, *P. syringae* (Ps), *P. graminis* (Pg), and *P. oryzihanitants* (Po), were analyzed for their ability to incite induced resistance. Since it was difficult to set up an experimental system suitable to analyze induced resistance using apple leaves and a specific apple pathogen, tobacco (*Nicotiana tabacum*, Xanthi-nc) and tobacco mosaic virus (TMV) were adopted as a model experimental system. "Xanthi-nc" is a tobacco variety with *N* gene, and when it is inoculated with TMV, it produces local necrotic lesions with hyper-sensitive reactions. Therefore, when induced resistance is activated in tobacco by *Pseudomonas* bacteria, TMV infection, i.e., formation of local lesions, should be inhibited.

Tobacco plants of the 12-leaf stage were evenly sprayed with 50 ml of *Pseudomonas* suspension on their fourth to ninth leaves, then sealed with a transparent plastic bag and grown in an incubation room controlled at 25°C, with 16 hours day-length. The assay was repeated twice, and each analysis consisted of three biological replicates. In the experiment, all *Pseudomonas* species were sprayed with the same colony formation unit (CFU) at 10^6/ml. Three days post application (dpa), leaves 4, 6, and 8 (from bottom to top) were cut off, dusted with carborundum (600 mesh), and rub-inoculated with 1 ml of purified TMV solution, with local lesion formation unit of ca. 50–200/ml per leaf. Inoculated leaves were placed in a transparent plastic container with a lid to keep the humidity moderate and incubated in the same incubation room under the same conditions. After 5 days the numbers of local lesions were counted and averaged. After 7 days of application, the same was repeated using leaves 5, 7, and 9.

In addition, in order to count the number of bacteria and to analyze the levels of

induced resistance elicited, two sets of two leaf disks (1 cm diameter) were punched out before TMV inoculation from leaves 4, 6, and 8 on 3 dpa, and from leaves 5, 7, and 9 on 7 dpa. These were combined into two sets (set 1 and set 2) of six leaf disks for each sampling day. Set 1 was used for counting the number of bacteria, and set 2 was used for total RNA extraction to examine activation of induced resistance response. Activation of induced resistance was assayed by analyzing the expression levels of three tobacco pathogenesis-related protein (PR protein) genes *ntPR1*, *ntPR2* (β-1,3-glucanase), and *ntPR3* (chitinase) as indicators. Total RNA was extracted using Trizol reagent (Invitrogen, Thermo Fisher Scientific Japan, Tokyo, Japan) according to the manufacturer's instructions, treated with RQ1 RNase-free DNase I (Promega Corporation, Wisconsin, USA), and cDNA was synthesized from 0.5 µg RNA as a template using Superscript VILO (Invitrogen). Analysis by qPCR was performed essentially as described in Kasai et al. [27] using THUNDERBIRD® Probe qPCR Mix (TOYOBO Life Science, Osaka, Japan) with a AriaMx realtime PCR system (Agilent Technology Inc., Tokyo, Japan). Information used to design the PCR primers for tobacco *ntPR1*, *ntRR2*, and *ntPR3* genes was obtained from methods reported in Zhang et al. [28] and Sun et al. [29]. The primers used for qPCR are described in Table SD1. The relative mRNA amounts were calculated by the $2^{-\Delta\Delta CT}$ method [30]. qPCR experiments were repeated twice, based on at least three biological replicates and three technical replicates. For statistical analysis, assuming the data obtained were normally distributed, an F-test was performed to check whether the data were evenly distributed. The calculation was performed using Excel, and it was assumed that the variance was unequal when the p value was 0.05 or less. Data assumed to be homoscedastic were checked for significant differences using Student's t-test, while those data assumed to be of unequal variances were checked for significant differences

using Welch's t-test. A two-sided test was performed using Excel, with a p value of 0.05 or less indicating a significant difference.

Meanwhile, for counting the number of bacteria, after punching out leaf disks, they were placed in a 50 ml Corning tube, added 10 ml of extraction buffer (0.85% NaCl, 0.01% Tween 20), and treated similarly as before to harvest bacteria on the leaf surface. Aliquots were spread on *Pseudomonas* selection medium to count the number of colonies. The experiment was repeated twice.

First, the same CFU of bacteria were sprayed on leaves 4 to 9 of tobacco plants, and TMV was inoculated on leaves 4, 6, and 8 at 3 dpa, and on leaves 5, 7, and 9 at 7 dpa. We found that leaves sprayed with Pg showed the strongest inhibition against TMV infection both at 3 and 7 dpa, [Figure SD1 (a)]. Weak levels of inhibition were observed on those sprayed with Ps and Po at 7 dpa, however, infection with TMV appeared to be promoted even on those at 3 dpa [Figure SD1 (a)]. In terms of the number of bacteria on the leaf surface, all three species showed roughly the same numbers on 3 and 7 dpa after the same CFU of bacteria were sprayed. This result indicated that tobacco leaves sprayed with Pg more strongly inhibited TMV infection than those with Ps and Po.

In keeping with the result of the infection inhibition experiment of TMV, levels of PR protein gene *ntPR3* mRNAs were significantly higher (at 1% significance level) in the leaves sprayed with Pg either on 3 or 7 dpa [Figure SD1 (b)]. Expression levels were also significantly higher (at 5% significance level) in those sprayed with Ps compared to water control but lower than those with Pg either on 3 and 7 dpa [Figure SD1(b)]. On the other hand, induction of *ntPR3* by Po was not observed, and higher expression levels of *ntPR1* and *ntPR2* genes were not stably detected after application of any of *Pseudomonas* species examined.

These results indicate that Pg in the phyllosphere is potentially superior in its ability to incite induced resistance, supported by induction of higher levels of mRNA of PR proteins, in particular *ntPR3* in Pg-treated leaves.

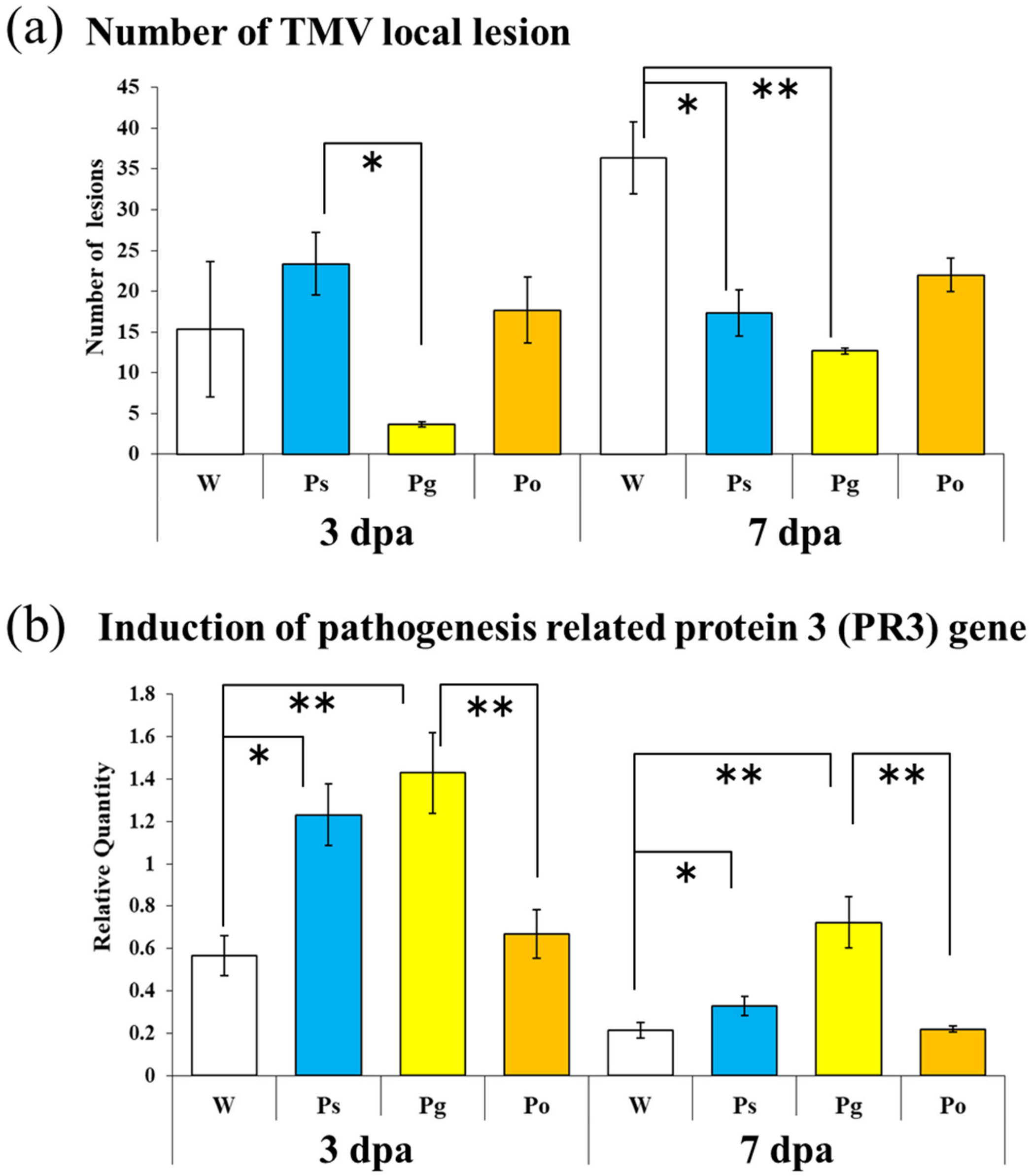

Figure SD1. Induction of resistance to TMV infection and activation of *ntPR3* gene expression by application of *Pseudomonas*. Tobacco plants (cv. Xanthi-nc) were evenly sprayed with *Pseudomonas* suspension on their fourth to ninth leaves, then challenge-

inoculated with TMV on leaves 4, 6, and 8 at 3 days post application (dpa), and leaves 5, 7, and 9 at 7 dpa. The assay was repeated twice, each analysis consisted of three biological replicates, and the numbers of local lesions were counted at 5 days after TMV inoculation. Lesions were significantly reduced by application of *P. graminis* (Pg) either at 3 and 7 dpa, and by P. syringae (Ps) at 7 dpa (a). In addition, before TMV challenge-inoculation, total RNAs were extracted for the analysis of expression of tobacco PR protein genes; i.e., *ntPR1*, *ntPR2*, and *ntPR3*, for RT-qPCR. Among three PR protein genes analyzed, the level of expression of ntPR3 was significantly higher on days 3 and 7 after Pg application. ntPR3 gene expression was also high in Ps, but the degree was small (b). Values with double asterisk (**; $p < 0.01$) and single asterisk (*; $p < 0.05$) were statistically significant at 1% and 5% levels, respectively, compared to water control or Pg.

Table SD1

Tobacco primers used for qRT-PCR

Gene	Name of Primer	sequence (5' to 3')
ntPR1	TPR1-F	GCTGAGGGAAGTGGCGATTTC
	TPR1-R	CCTAGCACATCCAACACGAACC
ntPR3	TPR3-F	GTGGTATGTTGAATGTTGCTCCTG
	TPR3-R	TGATCTAACGAATCCTAGCCTTGG
β-actin	β-actin-F	GAGGACAGGATGCTCCTCAG
	β-actin-R	AGACGCCTATGTGGGAGATG

Supplementary material References

27. Kasai, A.; Sano, T.; Harada, T. Scion on a stock producing siRNAs of potato spindle tuber viroid (PSTVd) attenuates accumulation of the viroid. *PLoS ONE* **2013**, *8*, e57736, doi:10.1371/journal.pone.
28. Zhang, J.Y.; Qiao, Y.S.; Lv, D.; Gao, Z.H.; Qu, S.C.; Zhang, Z. Malus hupehensis NPR1 induces pathogenesis-related protein gene expression in transgenic tobacco. *Plant Biol.* **2012**, *14* (Suppl. 1), 46–56, doi:10.1111/j.1438-8677.2011.00483.x.

29. Sun, T.; Liu, F.; Wang, W.; Wang, L.; Wang, Z.; Li, J.; Que, Y.; Xu, L.; Su, Y. The role of sugarcane catalase gene sccat2 in the defense response to pathogen challenge and adversity stress. *Int. J. Mol. Sci.* **2018**, *19*, 2686, doi:10.3390/ijms19092686.
30. Livak, K.J.; Schmittgen, T.D. Analysis of Relative Gene Expression Data Using Real-Time Quantitative PCR and the $2^{-\Delta\Delta CT}$ Method. *Methods* **2001**, *25*, 402–408, doi:10.1006/meth.2001.1262.
